# Dietary Emulsifier Sodium Stearoyl Lactylate Alters Gut Microbiota *in vitro* and Inhibits Bacterial Butyrate Producers

**DOI:** 10.3389/fmicb.2020.00892

**Published:** 2020-05-15

**Authors:** Lisa Elmén, Jaime E. Zlamal, David A. Scott, Ryan B. Lee, Daniel J. Chen, Alexandre R. Colas, Dmitry A. Rodionov, Scott N. Peterson

**Affiliations:** ^1^Tumor Microenvironment and Cancer Immunology Program, Sanford Burnham Prebys Medical Discovery Institute, La Jolla, CA, United States; ^2^Development, Aging and Regeneration Program, Sanford Burnham Prebys Medical Discovery Institute, La Jolla, CA, United States; ^3^A.A. Kharkevich Institute for Information Transmission Problems, Russian Academy of Sciences, Moscow, Russia

**Keywords:** microbiome, food additives, dysbiosis, short chain fatty acids, Western diet, bacterial genome reconstruction, proinflammatory

## Abstract

Dietary emulsifiers are widely used in industrially processed foods, although the effects of these food additives on human gut microbiota are not well studied. Here, we investigated the effects of five different emulsifiers [glycerol monoacetate, glycerol monostearate, glycerol monooleate, propylene glycol monostearate, and sodium stearoyl lactylate (SSL)] on fecal microbiota *in vitro.* We found that 0.025% (w/v) of SSL reduced the relative abundance of the bacterial class Clostridia and others. The relative abundance of the families *Clostridiaceae*, *Lachnospiraceae*, and *Ruminococcaceae* was substantially reduced whereas that of *Bacteroidaceae* and *Enterobacteriaceae* was increased. Given the marked impact of SSL on Clostridia, we used genome reconstruction to predict community-wide production of short-chain fatty acids, which were experimentally assessed by GC-MS analysis. SSL significantly reduced concentrations of butyrate, and increased concentrations of propionate compared to control cultures. The presence of SSL increased lipopolysaccharide, LPS and flagellin in cultured communities, thereby enhancing the proinflammatory potential of SSL-selected bacterial communities.

## Introduction

Emulsifiers are highly prevalent in the Western diet, however, little is known about how these compounds affect the structure and function of intestinal microbiota. Dietary emulsifiers are amphiphilic molecules that can bind water and lipids and are added to a wide range of industrially processed food products for the purpose of altering food consistency, enhance stabilization and shelf-life extension. A recent study investigating the dietary behaviors of the U.S. population found that 57.9% of the total energy intake by the study subjects was derived from ultra-processed foods, the food category that has the highest levels of artificial additives (Martinez Steele et al., 2016). Studies on populations with diverse food consumption habits and lifestyles have established that diet is the single largest influence on gut bacterial community composition and metabolic activity (Ley et al., 2008; De Filippo et al., 2010; David et al., 2014a, b, Schnorr et al., 2014; Smits et al., 2017). Western diet, defined as a diet high in sugar, protein and fat, accompanied by very low fiber intake, is a risk factor associated with multiple modern diseases (Okreglicka, 2015) and immigration studies have shown how U.S immigrants originating from developing nations over time and generations lose microorganisms they originally harbored (Vangay et al., 2018). This “modernization shift” has been proposed to be driven mainly driven by the lack of fiber in the Western diet (Sonnenburg et al., 2016; Vangay et al., 2018) but other factors, such as the specific impact and health implications of dietary emulsifier consumption remains understudied.

The U.S. Food and Drug Administration regulates food additives and specifies the amount that may be added to a given food product. Additives classified by the FDA as generally regarded as safe (GRAS), may be used in any amount. Traditional toxicology studies to date have not included analyses of the gut microbiota, and recent studies showed that two commonly used emulsifiers affect both host physiology and gastrointestinal bacteria (Chassaing et al., 2015; Viennois et al., 2017; Jiang et al., 2018). The emulsifiers carboxymethylcellulose (CMC) and polysorbate-80 (P80), which are both classified as GRAS, were shown to reduce the diversity of intestinal microbiota in both wild type and colitis susceptible mouse models. Exposure to CMC and P80 increased the relative abundance of taxa with the functional capability to degrade host glycans decorating mucin proteins, resulting in microbial encroachment of the mucin layer and a 50% reduction of mucous thickness. Increased proinflammatory bacterial LPS and flagellin were observed in serum, and gut permeability was enhanced as measured by serum antibodies to the aforementioned bacterial components. In addition, CMC and P80 treatment also induced dysbiosis and promoted low-grade inflammation and metabolic syndrome in wild type mice and severe colitis in IL-10^–/–^ mice (Chassaing et al., 2015). This study was extended to the AOM/DSS mouse model of colitis-associated cancer, in which the authors concluded that consistent with previous observations, P80 and CMC consumption enhanced tumor development (Viennois et al., 2017). Germ-free mice that received CMC- and P80-treated microbiota exhibited low-grade inflammation, whereas mice colonized with Altered Schaedler Flora showed no increase in inflammatory markers, indicating that emulsifier driven alterations in microbiota composition and activity contribute to inflammation (Chassaing et al., 2017). A mouse study on glycerol monolaurate similarly found evidence of changes to microbiota composition, low-grade inflammation and metabolic syndrome commensurate with increased serum LPS in low-fat diet fed mice (Jiang et al., 2018).

Given the widespread use of emulsifiers and the high intake of processed foods in the Western population it is important to gain a better understanding of the biological impact of emulsifiers in the diet. Single chain lipid amphiphiles, fatty acids, and derivatives such as monoglycerides are known to have antimicrobial properties (Kabara et al., 1972) and could potentially alter the composition of the intestinal microbiota and community metabolic activity. We have investigated the effects of five different dietary emulsifiers on gastrointestinal bacteria *in vitro*: glycerol monoacetate (AMG), glycerol monooleate (GMO), and glycerol monostearate (GMS) that are representatives of the widely used additive category mono- and diglycerides, and propylene glycol monostearate (PGMS) and sodium stearoyl lactylate (SSL), which are both common in bakery products, margarines, and desserts. Our results showed that 0.025% (w/v) of SSL, 10-fold lower than the FDA approved limit, significantly inhibits the bacterial families, *Clostridiaceae*, *Lachnospiraceae*, and *Ruminococcaceae*, and promote the expansion of *Bacteroidaceae* and proinflammatory *Enterobacteriaceae*. The presence of SSL also increased the level of LPS and flagellin in fecal bacterial cultures. Using genome reconstruction to analyze the capacity of the gut microbiome to express specific pathways, we predicted and confirmed by GC/MS analysis of short-chain fatty acids (SCFAs), that SSL exposure resulted in reduced production of butyrate.

## Materials and Methods

### Bacteria

Twelve healthy human subjects who each provided written informed consent donated stool samples collected in stool hats (Thermo Fisher Scientific). The study participants self-described as vegetarian, subjects with this diet were not specifically targeted. No detailed questionnaire accompanied sample collection and it is therefore not known if and to which extent the donors consumed foods containing any of the tested emulsifiers. The Institutional Review Board at Sanford Burnham Prebys Medical Discovery Institute approved the protocol (IRB-2014-020). The fecal samples were transferred to 50-ml conical tubes and stored at −80°C. To create a diverse inoculum for *in vitro* culture, the fecal samples were transferred on dry ice into a Coy Anaerobic chamber. The samples were thawed on wet ice to prevent bacterial growth and equal volumes of fecal material (using plastic spoons determined to contain a volume of 1 ml) were pooled and cold 1xPBS was added to make a total volume of 40 ml. The fecal suspension was vortexed and passed through a 70 μm filter to remove debris. The resulting filtrate was kept on wet ice and was mixed with glycerol (final concentration 25%) and aliquoted in cold cryotubes, frozen on dry ice and stored at −80°C until use.

The following bacterial species were purchased from Leibniz Institute DSMZ (Germany): *Blautia coccoides* (cat. 29138), *Clostridium methylpentosum* (cat. 5476) and *Roseburia intestinalis* (cat. 14610). *Clostridium clostridioforme* (cat. 25537) and *Enterococcus faecalis* (cat. BAA-2820) were purchased from ATCC (Manassas, VA, United States). *Escherichia coli ^pks^*^+^ was kindly shared by Christian Jobin, Ph.D. (University of Florida College of Medicine) and *Clostridium symbiosum*, *Faecalibacterium prausnitzii*, and *Lactobacillus casei* were kindly shared by Dan Peterson, Ph.D. (Johns Hopkins University).

### Fecal Bacterial Culture

A glycerol stock of frozen fecal bacteria, prepared from stool samples from twelve healthy human subjects (see the section “Bacteria” for details), was transferred on dry ice into a Coy Anaerobic chamber, and a scrape from this glycerol stock was suspended in 500 μl of 1xPBS and vortexed. 20 μl-volumes of the suspension were used to inoculate each tube (technical replicates) of 1 ml bacterial medium or bacterial medium supplemented with emulsifier. The glycerol stock was not thawed and was immediately returned to −80°C. Glycerol aliquots were discarded after three uses. Biological replicates followed the same procedure but started with a new scrape from a frozen fecal suspension glycerol stock and was performed on a different day. All fecal bacterial cultures were grown at 37°C in a Coy Anaerobic chamber, 9% H_2_ balance N_2_ without shaking. The media used for culture of the fecal bacteria were Brain Heart Infusion broth (BHI), BioWorld cat. 30620013-1, or chemically defined medium (CDM) with the following composition.

All chemicals used in CDM were purchased from Sigma-Aldrich (St. Louis, MO, United States) with the exception of hemin, which was purchased from Spectrum Chemical (New Brunswick, NJ, United States). The formulation of CDM contained 46 mM HEPES, cat. no. H3784, 1.7 mM KH_2_PO_4_, cat. no. P2222, 10.5 mM Na_2_HPO_4_, cat. no. S5761, 59.5 mM NaHCO_3_, cat. no S5761, and 100 mg/L each of guanine, cat. no. G11950, inosine, cat. no. I4125, thymidine, cat. no. T1895 and xanthine, cat. no. X3627. CDM also contained 100 mg/L choline chloride, cat. no. C7527, 500 mg/L ascorbic acid, cat. no. A4544, 2 mg/L lipoic acid, cat. no T1395 and 1.2 mg/L hemin, cat. no. H1003, dissolved in 0.2 M histidine, cat. no. H6034. 1 mg/L Resazurin, cat. no. R7017, was added to visually monitor dissolved oxygen. 11 g/L of Amino Acid Mix, cat. no. C0704, Teknova (Hollister, CA, United States), 10 mL/L each of Trace Mineral Supplement, cat. no MDTMS, and Vitamin Mix, cat. no. MD-VS, purchased from ATCC (Manassas, VA, United States). The pH of the medium was adjusted to 7.4 and was sterilized by filtration using a 0.22 μm filter flask. A mix of autoclaved α-cellulose, corn starch and inulin, were added to 2xCDM for a final concentration of 0.25% w/v of each complex carbohydrate in 1xCDM.

### Monoculture Growth Conditions and Media Formulations

Glycerol stocks with each bacterial species, stored in −80°C, were transferred into a Coy Anaerobic chamber on dry ice. A scrape from each glycerol stock, respectively, was suspended in 500 μl 1xPBS, vortexed, and 20 μl-volumes were used to inoculate five technical replicates of medium or medium with emulsifier for monoculture of each bacterium. Anaerobic bacteria were grown at 37°C without shaking in the anaerobic chamber. Growth curves were obtained by manual sampling followed by OD_600_ readings using a Synergy H1 Microplate reader, BioTek Instruments (Winooski, VT, United States). Aerobic bacteria were inoculated and grown in air, at 37°C without shaking and OD_600_ measurements were recorded automatically with the same instrument. Each growth experiment was repeated three times, starting with a new scrape from each glycerol stock, on a different day (biological replicates).

Bacterial monocultures were grown on the following medium formulations: Brain-Heart Infusion Broth (BHI), BioWorld cat.30620013-1 (*E. coli*, *E. faecalis*). Difco Lactobacilli MRS Broth, cat. 288130 (*L. casei*), Difco Reinforced Clostridial Medium (RCM), cat. 218081, (*B. coccoides*, *C. methylpentosum*, *C. clostridioforme*, *C. symbiosum*, and *R. intestinalis*). Peptone Yeast extract Glucose broth (PYG), originally formulated by Virginia Polytechnic Institute and State University (1977), was prepared in house for the culture of *F. prausnitzii.* Chopped meat medium no. 78, prepared according to DSMZ, was also used to culture *C. symbiosum.*

### Emulsifiers

Glycerol monoacetate, cat. no A14025, and GMS, cat. no. 43883 were purchased from Alfa Aesar (Heysham, England, United Kingdom). GMO, cat. no. NG-S235, was purchased from ChemService (West Chester, PA, United States). PGMS (35% monopalmitate), cat. no. P0614, was purchased from TCI America (Portland, OR, United States) and SSL 95%, cat. no. QJ-5146, was purchased from Combi Blocks (San Diego, CA, United States).

### 16S rRNA Sequencing

Fecal bacteria pools derived from 12 healthy donors were cultured as described above and 16S rRNA gene sequencing was used to analyze the relative abundance of five technical replicates per experimental group, BHI with or without emulsifier and CDM with or without emulsifier.

Bacterial DNA was extracted with the QIAamp DNA Mini Kit, cat. no. 51306 QIAGEN (Germany), according to the manufacturer’s instructions, with the addition of a 5-min bead beating step using a MiniBeadBeater, Biospec Products (Bartlesville, OK, United States), to ensure uniform lysis of bacterial cells. 16S rRNA gene libraries were generated according to Illumina’s 16S library preparation protocol with amplification of the V3–V4 region. Libraries were sequenced on the MiSeq instrument by Institute for Genomic Medicine, UCSD (La Jolla CA). Data were analyzed with CLC Workbench, Microbial module (QIAGEN, Germany). Each unique 16S rRNA gene sequence was subjected to BLAST using the NCBI 16S rRNA gene sequence database (Bacteria and Archaea) to identify best matches to taxa at the genus and species levels based on percent sequence identity.

### Low Range Inhibitory Concentration Test

Each bacterial species was inoculated by a scrape from frozen glycerol stocks, in a Coy Anaerobic chamber. The scrape was added to pre-reduced medium and was incubated at 37°C until the bacterial culture was cloudy, 24–72 h depending on bacterial species. A twofold dilution series was prepared in 96-well plates, 200 μl/well, starting with the highest concentrations at 0.025% or 0.0125% w/v and ending with the lowest concentration at 0.00078% w/v SSL. The plates containing media were pre-reduced for 24 h before inoculation with 10 μl-volumes of starter culture, in four to six wells per plate (technical replicates). The plates were covered with BreatheEasy film, cat. no. BEM-1, Diversified Biotech (Boston, MA, United States) and were incubated at 37°C until the wells with the lowest concentration of SSL showed signs of growth. At this point the plates were removed from the anaerobic chamber and shaken to create homogeneous suspensions of culture, followed immediately by OD_600_ measurements using a Synergy H1 Microplate reader, BioTek Instruments (Winooski, VT, United States). Uninoculated media control wells in columns parallel to the cultures were used to correct OD_600_ culture density data for different degrees of cloudiness caused by the different concentrations of SSL.

### Human TLR4/TLR5/NF-κB/SEAP Reporter HEK293 Cells

Engineered HEK293-reporter cells (HEK-Blue) cat. hkb-htlr4 and cat. hkb-htlr5, respectively, designed to monitor activation of human TLR4 and TLR5 and hence enable the detection of LPS and flagellin, respectively, were purchased from Invivogen, Inc. (San Diego, CA, United States). The cells were cultured in HEK-base medium: DMEM with 4.5 g/L glucose, cat. MT15017CV, Thermo Fisher Scientific (Waltham, MA, United States), 10% GemCell Fetal Bovine Serum, cat. 507532978, Thermo Fisher Scientific (Waltham, MA, United States), 50 U/50 μg/ml Penicillin/Streptomycin, cat. 30-002-Cl, 2 mM L-glutamine, cat. 25030-081, Thermo Fisher Scientific (Waltham, MA, United States) and 100 μg/ml normocin, Invivogen Inc. (San Diego, CA, United States).

The cells were supplied with selective antibiotics first after two passages to prevent cells from detaching from the culture dish, according to the manufacturer’s instructions. Cells were approximately 70% confluent when removed for each assay. Cells were gently removed by pipetting small volume of 1xPBS, counted in a Bio-Rad cell counter and diluted in HEK-detection medium cat. hb-det, Invivogen Inc. (San Diego, CA, United States) added to 140,000 cells/ml. Centrifugation was avoided as this stressor can confound the reporter readout.

Fecal bacteria were inoculated and cultured anaerobically as described above in the section Fecal bacterial culture, but in CDM without resazurin to prevent any confounding effects on the colorimetric SEAP reporter. Three to six technical replicates of overnight fecal bacterial cultures with or without SSL were diluted in a twofold dilution series to avoid overloading the reporter, and 20 μl-volumes of each sample and dilution step was added to reporter cells suspended in HEK-detection medium, 180 μl/well in a clear, flat bottom 96-well plate. Standard curves were generated by stimulating reporter cells with a dilution series (10^1^–10^4^ ng/ml) of either LPS, LPS-EB Ultrapure, cat. tlrl-3pelps, or Flagellin, FLA-ST cat. trl-stfla, both purchased from Invivogen, Inc. (San Diego, CA, United States). Ultrapure water, cat. 18-193 Genesee Scientific Corp., (San Diego, CA, United States), was used as negative control. The 96-well plates were incubated at 37°C in a cell incubator with 5% CO_2__._ OD_655_ was recorded after 7–10 h of stimulation, and the results were normalized by OD_600_ of the corresponding bacterial culture sample. The concentrations of LPS and flagellin were calculated based on the standard curve for each experiment. Three biological replicates were performed, with new fecal bacterial overnight cultures and new passages of reporter cells for each experiment.

Fold change was calculated by dividing the measured concentration of LPS or flagellin with the average concentration of LPS or flagellin in the control samples (bacterial culture in medium without emulsifier). Figure 4 represents all three biological replicates.

HEK-reporter cell viability was determined by counting viable cells after 8 h of exposure to culture media and bacterial cultures. This was done by preparing cells as described in preparation for the colorimetric SEAP assay, with 20 μl of uninoculated medium or bacterial culture added to 180 μl HEK-cell suspension (140,000 cells/ml) in HEK-base medium. After 8 h of exposure, 10 μl of cell suspension was mixed 1:1 with Trypan Blue, and 10 μl was dispensed into a Luna Cell Counting slide, cat. no. L12001, and counted in a Luna II Automated Cell Counter, Logos Biosystems (South Korea). No statistically significant difference in cell viability was observed between the different conditions.

### Genome Reconstruction of SCFA Pathways

Short-chain fatty acids are end-products of anaerobic fermentation of dietary carbohydrates by intestinal microbiota. To predict metabolic capabilities of microbial taxa identified by 16S rDNA analysis, we performed genomics-based reconstruction of metabolic pathways for butyrate and propionate synthesis. We used a subsystems-based approach implemented in the SEED genomic platform (Overbeek et al., 2014) to capture, analyze and extend enzymes involved in SCFA metabolism in >2,200 microbial genomes. The reference set of 2,228 genomes representing ∼700 microbial species from human gut was previously assembled from the literature (Rodionov et al., 2019). The metabolic subsystems were developed and propagated based on previous studies of phylogenetic distribution of bacterial pathways for production of butyrate (Vital et al., 2014) and propionate (Reichardt et al., 2014) (Supplementary Figure S4). As a result, each reference genome in each analyzed subsystem was assigned a binary (“1” or “0”) phenotype reflecting the presence/absence of at least one functional pathway variant, as both propionate and butyrate subsystems include four different pathway variants. The obtained binary phenotype matrix (BPM) for reference genomes was used to calculate a community phenotype matrix (CPM) for all mapped taxa obtained from 16S analysis by averaging the respective CPM values as previously described (Rodionov et al., 2019). The community phenotype index (CPI) for each 16S sample was calculated as the sum of the respective CPM values of each taxon multiplied by their relative abundances. CPI gives a probabilistic estimate of the fraction of cells (on the scale 0–100%) in the community possessing a specific metabolic pathway.

### Determination of Propionate Producing *Escherichia* by PCR

DNA extracted from five different CDM cultures enriched for *Escherichia* as determined with 16S rRNA gene sequencing, and DNA extract from the control culture *E. coli* BW25113, were quantified with NanoDrop (Thermo Scientific), and 10 ng DNA template was used for each PCR reaction. PCR was performed on a Mastercycler Pro thermocycler (Eppendorf) using Phusion High-Fidelity DNA Polymerase (New England Biolabs) and primers designed to target *Escherichia-*specific genes: the universal *Escherichia* genes *nadE* and *ribF* and propionate-producing genes *pduC* and *pduP* (Supplementary Table S1). PCR reactions for *ribF* and *pduC* genes were run in the same program simultaneously to maintain identical conditions (Supplementary Table S2). 5 μL of each PCR product was loaded on a 1.5% TAE-agarose gel, 5 μL of Quick-Load Purple 100 bp DNA Ladder (New England BioLabs, Ipswich, MA, United States) was loaded in the center of the gel for quantification and size comparison.

### GC-MS Analysis of SCFA Concentrations

Fecal bacteria were inoculated and cultured in CDM as described above, with four to five technical replicates per experimental group. Sample aliquots were taken from the cultures at late exponential phase and early lag phase. Culture density was determined by OD_600_ measurements using a Synergy H1 Microplate reader, BioTek Instruments (Winooski, VT, United States). The samples were centrifuged to pellet the bacterial cells and stored in −80°C until the culture supernatant was analyzed by GC-MS. Two biological replicates, defined as a new inoculum cultured on a different day, were performed.

The analysis of SCFA was modified from the method of Hoving et al. (2018). Medium samples (2 μl, from four to five cultures, technical replicates of each sample type) were diluted with water to 40 μl and mixed with 10 μl internal standards mix (100 μM each D3-acetate, D5-propionate, D7-butyrate, and 2-ethylbutyrate, all from Sigma) and 2.5 μl 0.5 M NaOH. A range of 8 standards containing 0.1 to 20 nmol each of 10 SCFAs: acetic acid, butyric acid, formic acid, heptanoic acid, hexanoic acid, isobutyric acid, isovaleric acid, 4-methylvaleric acid, propionic acid, and valeric acid (Supelco Volatile Free Acid Mix, cat. no. CRM46975, Sigma) and the above internal standards was prepared in parallel. Pentafluorobenzyl bromide (Sigma) (100 μl × 172 mM, in GC-grade acetone, Fisher) was added to samples and standards, and these were incubated on a rotating hotplate for 30 min at 60°C and 650 rpm. After cooling to room temperature, water (750 μl) and chloroform (60 μl) were added. Tubes were vortexed briefly, twice, and centrifuged to separate the phases (15,000 × *g*, 5 min, 4°C). The lower chloroform phase was transferred to GC-MS vial inserts.

Samples and standards were analyzed using a Shimadzu QP-2010 Plus GC-MS with a Rxi-5ms column (15 m × 0.25 i.d. × 0.25 μm, Restek). The GC-MS was programmed with an injection temperature of 250°C, 1.0 μl injection volume and 1/10 split ratio. The GC oven temperature was initially 70°C for 4 min, rising to 160°C at 6°C/min, and to 280°C at 50°C/min with a final hold at this temperature for 2 min. GC flow rate, with helium as the carrier gas, was 50 cm/s. The GC-MS interface temperature was 300°C and (electron impact) ion source temperature was 200°C, with 70 eV ionization voltage. Mass fragments in the m/z range 43–400 were scanned (fragments of m/z 181 and 240 were used for acetate; m/z 46 for D3-acetate; m/z 57 and 254 for propionate; m/z 62 and 259 for D5-propionate; m/z 181 and 268 for butyrate; m/z 50 and 78 for D7-butyrate; m/z 282 for isovalerate; m/z 43, 71, 181 and 268 for 2-ethylbutyrate; other SCFA were not detectable). Data from standards were used for calibration in MetaQuant (Bunk et al., 2006), taking average values where more than one fragment was used, and metabolite amounts were adjusted for recovery of the deuterated internal standards (2-ethylbutyrate was used as internal standard for isovalerate).

### Statistical Analyses

Principal coordinates plots were based on phylogenetic trees constructed by CLC Workbench, Microbial module, and distances were calculated using the UniFrac distance metric (Lozupone et al., 2011). LDA effect size, LefSe (Segata et al., 2011), which combines Kruskal–Wallis with Linear Discriminant Analysis effect size, was used to determine statistically significant shifts in abundance of bacterial families. One-way ANOVA with Tukey’s multiple comparisons test and two-tailed Student’s *t*-test was used in in Graph Pad Prism as appropriate to establish statistical significance for differences in bacterial abundance at the genus level.

## Results

### Emulsifiers GMS and SSL Affect Bacterial Community Composition

To create a highly diverse inoculum of fecal bacteria, we combined stool samples from 12 healthy, vegetarian subjects (U.S. residents), and used this pool of bacteria (Supplementary Figure S1A) for the subsequent culture experiments. In order to establish whether any of the selected emulsifiers affected bacterial community composition *in vitro*, we cultured the fecal bacteria in a rich medium (BHI) supplemented with each emulsifier. We based the experimental concentration of emulsifiers on the only FDA-regulated emulsifier, SSL, (maximum 0.2% or 0.5% in weight of finished product) and reduced the concentration by approximately 10-fold, to 0.025% (w/v), to account for variability in manufacturer practices and the likely dilution by consumption of non-emulsifier containing foods.

**FIGURE 1:**
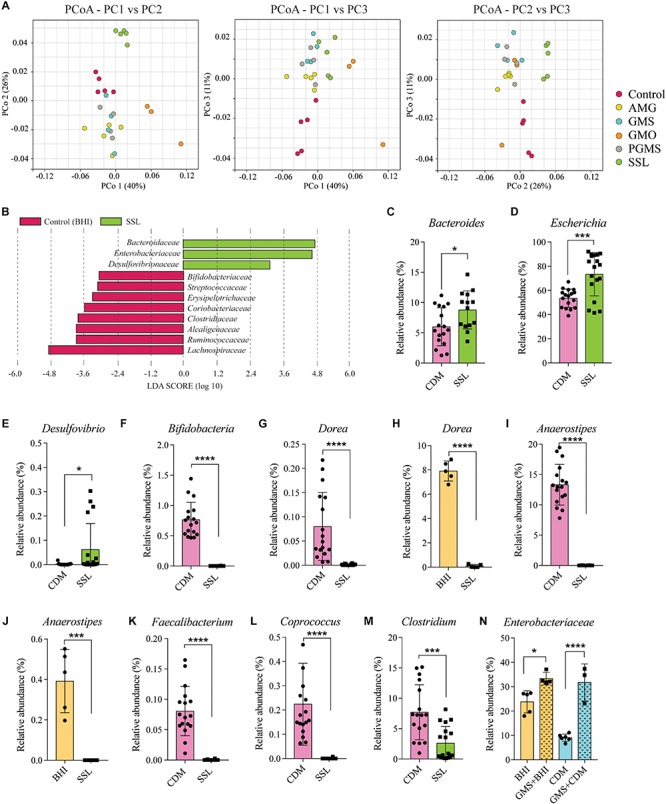
Exposure to dietary emulsifiers SSL and GMS alter gut microbiota composition *in vitro* independent of medium formulation. **(A)** Principal coordinates analysis (PCoA) of β-diversity based on the distance metric unweighted UniFrac of control fecal bacterial cultures and those exposed to 0.025% of the emulsifiers AMG, GMS, GMO, PGMS, and SSL. Cultures exposed to SSL cluster separately from controls. **(B)** LefSe (Segata et al., 2011) analysis determined which bacterial families that were significantly altered by SSL. *Bacteroidaceae*, *Enterobacteriaceae*, and *Desulfovibrionaceae* (green bars) were enriched in SSL-cultures, and multiple families including *Ruminococcaceae* and *Lachnospiraceae* (red bars) were reduced in abundance. **(C–E)** *Bacteroides*, *Escherichia* and *Desulfovibrio* display increased relative abundance in the presence of SSL in chemically defined medium (CDM). **(F–M)** Genera decreased in relative abundance in the presence of SSL (CDM). **(N)** GMS exposure increased the relative abundance of *Enterobacteriaceae* across medium types (*n* = 5, one biological replicate per medium type). Statistical significance of pairwise comparisons was determined with two-tailed Student’s *t*-test or one-way ANOVA with Tukey’s multiple comparisons, as appropriate, using Graph Pad Prism *<0.05, **<0.01, ***<0.001, ****<0.0001.

Results of sequencing and analysis of the V3–V4 region of the 16S rRNA gene of bacteria cultured in media containing the selected emulsifier were compared to that for bacteria grown in media without emulsifier to identify changes in the relative abundance of taxa present in fecal communities. We note that >60% of the taxa observed in the fecal pool inoculum were cultivable, representing the majority of genera and species present in fecal microbiota. The β-diversity of communities grown in BHI with and without emulsifier was compared by principal coordinates analysis (PCoA), which revealed that cultures exposed to all emulsifiers clustered separately from control samples (Figure 1A). We noted that SSL, PGMS, GMS, and GMO supplementation of BHI resulted in increased relative abundance of *Enterobacteriaceae* (Figure 1B and Supplementary Figure S1B). Additional shared responses to emulsifier supplementation were evident including the reduced relative abundance of *Erysipelotrichaceae* in SSL, PGMS, GMS and GMO (Figure 1B and Supplementary Figure S1C).

We next repeated the experiments with a CDM to determine if the effect of SSL was independent of medium-specific effects. While each emulsifier modulated fecal communities in both CDM and BHI, only SSL exerted the same effects independent of the media tested. We therefore focused additional analyses on SSL. At the family level, SSL supplementation drove significant increases in the relative abundance of *Bacteroidaceae*, *Enterobacteriaceae*, and *Desulfovibrionaceae* and a significant reduction in the relative abundance of multiple families within the class Clostridia including *Clostridiaceae*, *Ruminococcaceae*, and *Lachnospiraceae* (Supplementary Figure S1D).

The increased relative abundance of families *Bacteroidaceae* and *Enterobacteriaceae* observed in both SSL-supplemented medium types were mainly, but not exclusively, due to expansion of genera *Bacteroides* and *Escherichia* respectively (Figures 1B–D and Supplementary Figures S1E,F). *Desulfovibrio* was the only genus identified belonging to *Desulfovibrionaceae* and thus was solely responsible for the increase in the relative abundance of this family (Figure 1E and Supplementary Figure S1G). Likewise, a significant reduction in *Bifidobacteriaceae* was due to a suppression of the genus *Bifidobacterium* (Figure 1F and Supplementary Figure S1H). The genera responsible for the reduction of Clostridia were consistent between BHI and CDM in terms of direction, but variability in the relative abundance was noted, (e.g., *Dorea* and *Anaerostipes*, Figures 1G–J) and likely due to the differences in the nutrient composition of the medium. *Dorea, Anaerostipes, Faecalibacterium*, and *Coprococcus* (Figures 1H–L and Supplementary Figures S1I,J), as well as *Flavonifractor* and *Pseudoflavonifractor* (Supplementary Figures S1K,L) were all suppressed to the limit of detection and *Clostridium* spp. were reduced by twofold (Figure 1M and Supplementary Figure S1M). *Blautia* was also significantly reduced by SSL but only in CDM-cultures (Supplementary Figure S1N). The population of *Lachnoclostridium* spp. was highly abundant in BHI-culture (Supplementary Figure S1O), but significantly reduced in SSL-supplemented BHI cultures. This taxonomic group displayed low relative abundance in CDM-culture and showed no significant change by SSL (Supplementary Figure S1P). Among the other emulsifiers tested, only GMS had a consistent effect that was independent of medium type and resulted in increased relative abundance of *Enterobacteriaceae* (Figure 1N). These results demonstrate that 0.025% (w/v) SSL has a medium-independent, negative effect on the abundance of several genera in the class Clostridia.

### Representative Clostridial Species Are Inhibited by SSL

To determine whether the reduced relative abundance of bacterial species belonging to the families *Clostridiaceae*, *Lachnospiraceae*, and *Ruminococcaceae* was the result of sensitivity to SSL or due to indirect effects arising from the increased relative abundance of other taxa in the communities, we selected two representative organisms for each family for evaluation in pure culture. *Roseburia intestinalis* and *Faecalibacterium prausnitzii* are both organisms associated with health (Martin et al., 2017; Tamanai-Shacoori et al., 2017) and their corresponding genera were abundant in the fecal inoculum (Supplementary Figure S1A). We also selected *C. symbiosum*, the most abundant *Clostridium* species in the fecal cultures, together with the low abundance *C. clostridioforme*, and included *Blautia coccoides* and *C. methylpentosum* to extend our observations to additional representative species. We selected *Escherichia coli*, *Enterococcus faecalis* and *Lactobacillus casei* to serve as control organisms (Table 1).

**TABLE 1:** Representative isolates of bacterial families.

**Family**	**Bacterium**
*Clostridiaceae*	*Clostridium clostridioforme*
	*Clostridium symbiosum*
*Lachnospiraceae*	*Blautia coccoides*
	*Roseburia intestinalis*
*Ruminococcaceae*	*Clostridium methylpentosum*
	*Faecalibacterium prausnitzii*
*Enterobacteriaceae*	*Escherichia coli ^pks+^*
*Enterococcaceae*	*Enterococcus faecalis*
*Lactobacillaceae*	*Lactobacillus casei*

**FIGURE 2:**
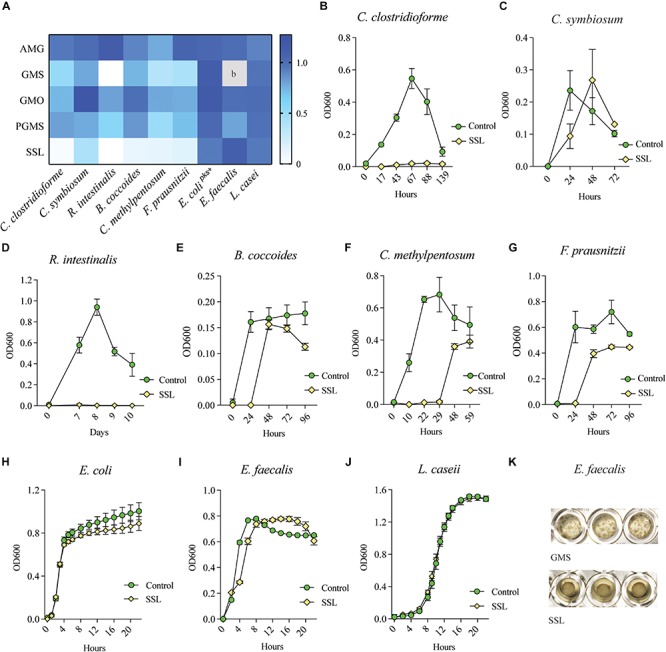
SSL inhibits growth of Clostridial species in monoculture. **(A)** Heatmap illustrating the sensitivity of Clostridial species to emulsifiers GMS, PGMS and SSL as compared to control organisms *E. coli*, *E. faecalis* and *L. casei*. 0 = emulsifier inhibited growth, 1 = growth equal to control culture. b = *E. faecalis* formed a biofilm in response to GMS in all experiments (technical replicates *n* = 6, biological replicates *n* = 3–4). Colors represent the mean ratio of three biological replicates (*n* = 15). **(B–G)** Representative bacterial growth curves show that SSL is inhibitory to all tested Clostridial species, and potentially bactericidal to *C. clostridioforme* and *R. intestinalis*. **(H–J)** Control organisms *E. coli*, *E. faecalis* and *L. casei* were unaffected by SSL. **(K)** *E. faecalis* adhered to well bottom in response to GMS in all experiments and in half of SSL experiments.

Bacteria were cultured in aerobic or anaerobic conditions as appropriate, in rich medium with or without 0.025% (w/v) of emulsifier, and growth was monitored with OD_600_ absorbance measurements. The ratio of bacterial culture density, calculated as emulsifier culture/control medium culture at their respective peak OD_600_ value, is summarized in Figure 2A (*n* = 3–6 technical replicates, 3–4 biological replicates). Most species belonging to Clostridia were sensitive to SSL and showed varying degrees of inhibition by both GMS and PGMS, while the growth of *E. coli*, *E. faecalis* and *L. casei* was largely unaffected (Figures 2A,H–J). With the exception of *C. clostridioforme* (Figure 2B) and *R. intestinalis* (Figure 2D), which were completely inhibited in SSL-supplemented medium after more than one week, the other Clostridia tested displayed a significant SSL-dependent lag in growth, but these microbes were eventually able to reach reasonably high densities (Figures 2E–G). After the lag phase, which varied from hours to multiple days depending on the bacterium, cultures entered an exponential phase and, in the case of *C. symbiosum*, surpassed the density of the control culture (Figure 2C). *E. faecalis* formed a biofilm in response to SSL in two out of four experiments, which prevented collection of reliable culture density remeasurements (Figure 2K). The experiments in which bacterial cells remained in suspension are depicted in the heatmap (Figure 2A).

Glycerol monoacetate (AMG) increased the growth of all tested bacteria to some extent, with the exception of *C. methylpentosum*, which showed slightly reduced growth (Figure 2A). GMS negatively impacted the growth of all Clostridial isolates, ranging from strong (*R. intestinalis*) to a moderate inhibition (*C. symbiosum* and *B. coccoides*) (Figure 2A and Supplementary Figures S2A–H). *E. coli* and *L. casei* were not affected by GMS (Figure 2A and Supplementary Figures S2G,I), while in all four experiments *E. faecalis* formed a biofilm Figure 2K) that adhered to the well and prevented comparison of OD_600_ values with other culture conditions. GMO promoted a small increase in growth of *C. symbiosum*, whereas all other Clostridia showed some degree of growth inhibition (Figure 2A). The population of *E. coli* increased slightly, while *E. faecalis* and *L. casei* were unaffected by GMO. PGMS-supplemented cultures displayed variable inhibitory effects on the growth of all bacteria, with the exception of *E. coli* and *L. casei* (Figure 2A and Supplementary Figures S2G,I).

Based on these results, we conclude that 0.025% SSL (w/v) is inhibitory to four of six tested Clostridial species and likely bactericidal to *C. clostridioforme* and *R. intestinalis*. GMS and PGMS also suppressed growth of all tested species, albeit to a lesser extent than SSL.

### Low Concentrations of SSL Are Bactericidal and Inhibitory to Clostridial Species

**FIGURE 3:**
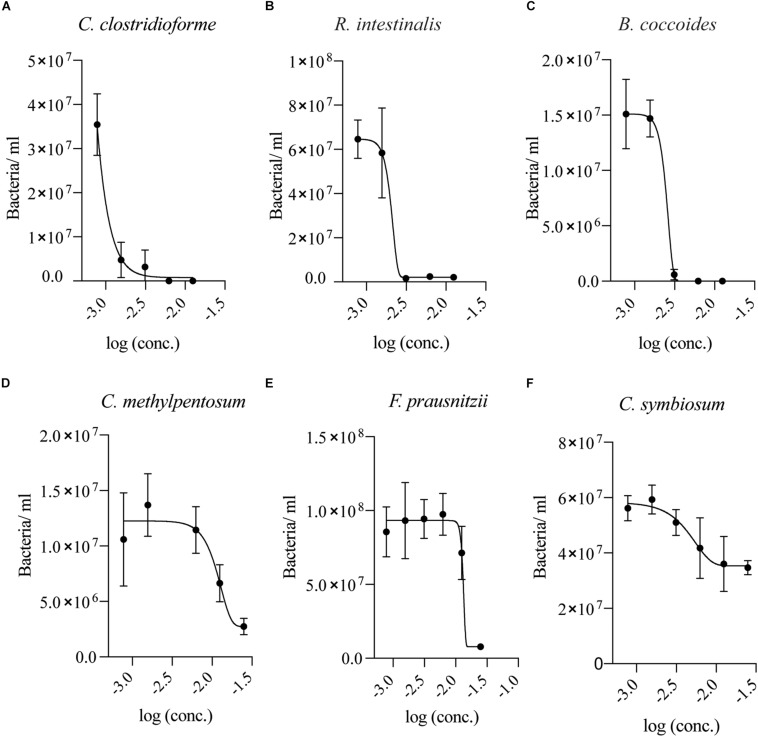
Low concentrations of SSL inhibits Clostridial species. Growth of **(A)** *C. clostridioforme*, **(B,C)** *R. intestinalis* and *B. coccoides*, and **(D)** *C. methylpentosum* growth was suppressed at SSL concentrations >0.0015% (log –2.8), >0.003% (log –2.5), and >0.01% (log –2) (w/v), respectively. **(E)** *F. prausnitzii* was inhibited at concentrations >0.013% (log –1.9) (w/v) SSL. **(F)** *C. symbiosum* had the lowest sensitivity of the tested bacteria and grew at 0.025% but growth was suppressed (*n* = 18, three biological replicates).

Since two of the tested bacterial species showed no signs of growth in the presence of SSL after multiple days, a twofold dilution series of SSL in appropriate growth media for each isolate was established to determine if concentrations lower than 0.025% were inhibitory. For the most sensitive organisms, *C. clostridioforme* and *R. intestinalis*, SSL concentrations >0.0015% SSL (w/v) inhibited growth, whereas *B. coccoides*, was inhibited by >0.003% SSL (w/v) (Figures 3A–C). *C. methylpentosum* displayed impaired growth at 0.025% (w/v) SSL but exhibited normal growth at concentrations <0.01% SSL (w/v) (Figure 3D). *F. prausnitzii* growth was inhibited at concentrations >0.013% (w/v) SSL (Figure 3E). *C. symbiosum* was the least sensitive organism tested and could grow in the presence of 0.025% (w/v) but growth was suppressed (Figure 3F). It is notable that SSL sensitivity curves for most species show marked growth inhibition at discrete inhibitory concentrations as reflected by the slope of the inhibition curves.

### SSL Increases Expression of LPS and Flagellin in Culture

**FIGURE 4:**
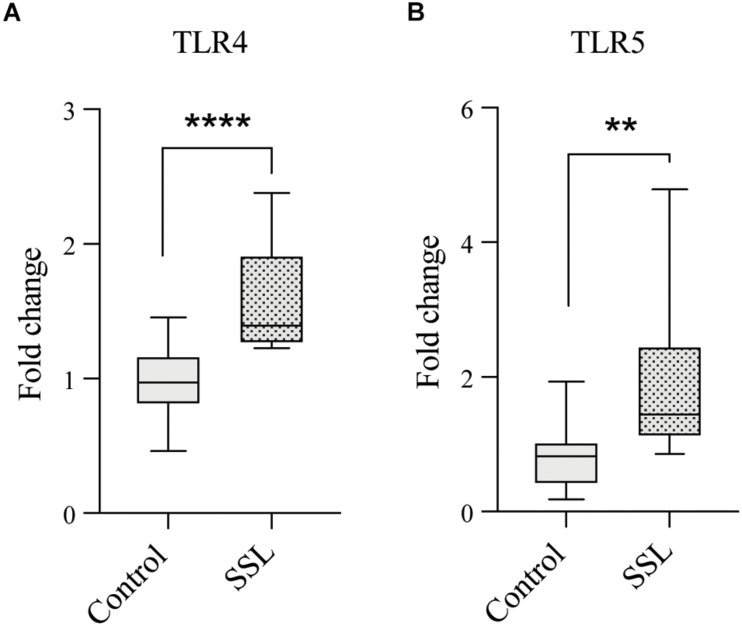
SSL exposure increased levels of proinflammatory bacterial products. Culture of fecal bacteria in SSL-supplemented medium resulted in increased levels of **(A)** Gram-negative cell wall component lipopolysaccharide (LPS) and **(B)** Flagellin, a flagellum component, measured by colorimetric indicator SEAP in engineered HEK-Blue reporter cells expressing TLR4 and TLR5, respectively (*n* = 15, three biological replicates). Statistical significance of pairwise comparisons was determined with a two-tailed Student’s *t*-test, using Graph Pad Prism *<0.05, **<0.01, ***<0.001, ****<0.0001.

Western diet is associated with increased intestinal barrier permeability and endotoxemia, due to high plasma levels of the Gram-negative cell wall component lipopolysaccharide (LPS), which can cause systemic inflammation (Pendyala et al., 2012). Emulsifiers have been shown to reduce barrier integrity (Chassaing et al., 2015) and since SSL exposure increased the abundance of *Escherichia*, a source of both LPS and flagellin, we next evaluated the proinflammatory potential of SSL-supplemented cultures by measuring human Toll-like receptor 4 (TLR4) and 5 (TLR5) activation by LPS and flagellin, respectively, using engineered HEK-Blue reporter cells (Invivogen Inc., San Diego). Both TLR4 and TLR5 consistently showed a higher degree of activation in response to SSL-supplemented bacterial cultures (1.6- and 1.9-fold increase respectively) compared to those without SSL (Figures 4A,B), indicating that SSL promotes an increase in bacteria expressing proinflammatory components.

### Butyrate Production Is Decreased by SSL Exposure

**FIGURE 5:**
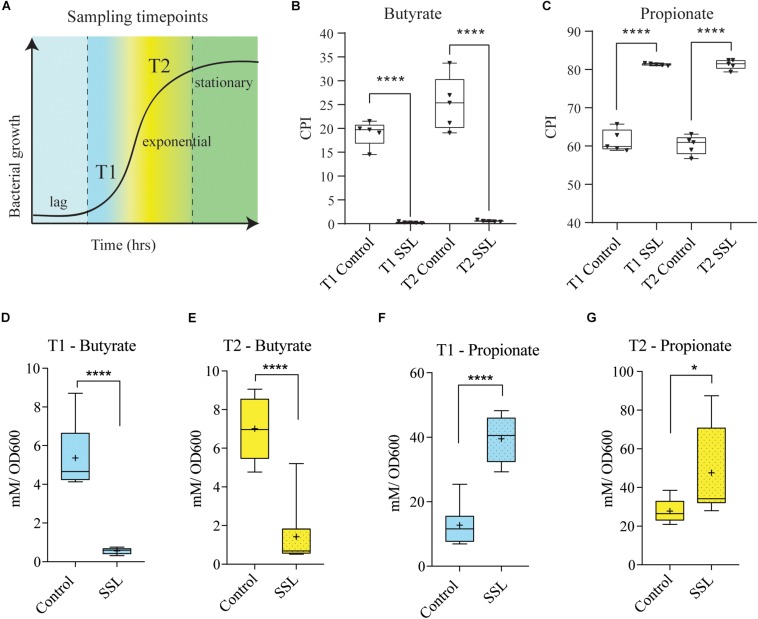
SSL reduces the capacity of the bacterial culture to produce butyrate. **(A)** Schematic of experimental design. Sampling timepoint T1 and T2 represents early log phase and the end of exponential phase, respectively, in cultures of fecal bacteria in CDM. **(B)** Community phenotype index (CPI) shows the percentage of bacterial community members that encoded complete butyrate pathways, predict that control cultures at both timepoints produce significantly more butyrate (20%, 25% butyrate producers) than the SSL-treated cultures (<1% of butyrate producers). **(C)** CPI showing the percentage of bacterial community members that encode complete propionate pathways, predict that SSL-treated cultures (>80%) at both timepoints produce significantly more propionate than controls (∼60%). **(D,E)** Butyrate production as measured by GC-MS was significantly higher in control cultures compared to SSL-treated cultures at both T1 and T2. **(F,G)** Propionate production as measured by GC-MS was significantly lower concentration in control cultures relative to SSL-treated cultures at both T1 and T2. Bacterial culture experiments and SCFA analysis: *n* = 9–10, two biological replicates. Statistical significance of pairwise comparisons was determined with a two-tailed Student’s *t*-test, using Graph Pad Prism *<0.05, **<0.01, ***<0.001, ****<0.0001.

To evaluate butyrate production in control and SSL-supplemented communities, we repeated the fecal cultures in CDM and sequenced the V3–V4 region of 16S rRNA gene derived from these communities. As observed previously, the relative abundance of many bacterial species in the families *Clostridiaceae*, *Lachnospiraceae* and *Ruminococcaceae* were reduced by SSL. It is known that these families are enriched in species capable of producing butyrate, therefore we asked whether butyrate producing microbes are negatively impacted by SSL. Initially, we used genome reconstruction based on reference genomes corresponding to those observed in each culture to predict butyrate and propionate production capacity. The presence of one or more of the four known pathways encoding butyrate and propionate biosynthetic capacity (Supplementary Figure S4) was scored for each taxon observed in 16S rRNA gene profiles (see the section “Materials and Methods” for a complete description). The control cultures were predicted to contain a significant fraction of butyrate producers relative to SSL cultures at both sampling time points (Figure 5A). The mean CPI for control cultures was 20% (T1) and 27% (T2) respectively, whereas SSL-supplemented cultures were predicted to dramatically reduce the total proportion of butyrate producing microbes to ∼1% of the total community. By contrast, SSL-supplemented cultures were predicted to strongly increase the relative abundance of propionate producers in communities (Figure 5B).

To verify these predictions, we analyzed culture supernatants derived from the same fecal communities by GC-MS to measure SCFA concentrations. Culture aliquots were removed at the start and end of exponential phase to obtain SCFA measurements at two different time points (Figure 5C). The butyrate concentration was significantly reduced at both timepoints in the SSL treated cultures, with a 10-fold decrease at T1 and a 5-fold decrease at T2 (Figures 5D,E). As predicted, the propionate concentration was significantly increased in the SSL-cultures, with a 3-fold difference observed at T1 and a 1.7-fold difference at T2 (Figures 5F,G). Predicted phenotypes correlated very well with the measured concentrations of butyrate and propionate (Supplementary Figures S3A–D). Acetate production tended to be higher in the SSL-exposed culture, at both T1 and T2, however, the difference was not statistically significant over biological replicates (Supplementary Figures S3E,F). The high concordance between *in silico* predicted and experimentally measured butyrate and propionate provide a strong validation of the genome reconstruction methods to predict butyrate and propionate biosynthetic potential of complex communities.

A complication encountered for genome reconstruction of propionate biosynthetic potential involved the SSL-dependent enrichment of *Escherichia*. Within this genus some species encode propionate pathway genes, whereas others do not. Due to the high similarity of 16S rRNA gene sequence amongst *Escherichia* species, we could not distinguish between *E. coli* that does not produce propionate and the related propionate producer, *E. fergusonii*. Therefore, we analyzed the genomic DNA of the cultures by PCR, using *Escherichia*-specific primers targeting *ribF*, and *Escherichia-*specific primers targeting the propionate enzyme encoding gene *pduC*. The ratio of *pduC*/*ribF* was 0.68–0.89 in the culture samples and zero for the reference bacterium *E. coli* (Supplementary Figure S3C), indicating that the majority of *Escherichia* in our cultures could produce propionate, and were likely representative of the species *E. fergusonii*. It should be noted that SSL-dependent increases in propionate production are also strongly attributed to expansion of *Bacteroides* in SSL-supplemented cultures, since members of this genus encode propionate biosynthetic potential.

In summary, these experiments show that emulsifiers alter the composition of gut bacterial communities *in vitro* and that the growth-suppressing effect of SSL on Clostridia negatively affected butyrate production and increased propionate production.

## Discussion

In this study, we investigated the effects of common dietary emulsifiers on the composition of fecal microbiota *in vitro*. A significant advantage of studying the effects of emulsifiers *in vitro* is the ability to distinguish direct microbiological effects in the absence of complicating host-dependent effects. Among the emulsifiers tested, we found that the impact of SSL was media-independent and therefore focused our analysis on this emulsifier. We found that low concentrations of SSL significantly inhibited growth of bacteria in the class Clostridia, which was accompanied by the expansion of the families *Bacteroidaceae* and *Enterobacteriaceae.* Given these observed alterations in microbiota composition, we used genome reconstruction methods to predict the capacity of the resulting communities to produce butyrate and propionate. The dramatic changes in SCFA production capacity prompted us to experimentally verify these predictions, which were in strong agreement with *in silico* predictions. SSL-supplemented cultures had decreased butyrate production and increased propionate production. In addition, SSL exposure enhanced the proinflammatory potential of the bacterial community due to increased levels of LPS and flagellin. We acknowledge that *in vitro* studies of gastrointestinal bacteria have limitations, since the lack of host factors and medium formulations tend to favor the expansion of specific taxa, as observed here with *Escherichia*. However, while the cultured composition of fecal bacteria does not quantitatively reflect the normal composition of the human microbiome, it nevertheless revealed significant differences between control and emulsifier treated conditions and prompted us to investigate and validate the suppressive effect of SSL using monocultures of bacteria representative of affected families.

Experiments with pure cultures of representative bacterial strains showed that all tested bacteria tolerated AMG and GMO, while GMS, PGMS, and SSL all inhibited growth of Clostridia to varying extents. SSL exerted the strongest effect, but since GMS and PGMS also suppressed Clostridial growth we hypothesized that structural features of these emulsifiers, i.e., the stearate component, a saturated fatty acid with an 18-carbon chain, may be a determinant of their microbiological effects (Supplementary Figure S3H). Lauric acid (a 12-carbon chain) has been shown to have potent antimicrobial activity against Gram-positive organisms, an activity that is increased in its glycerol derivative form, glycerol monolaurate (Yoon et al., 2018). However, *L. casei* and *E. faecalis* were not affected by GMS, PGMS or SSL in our experiments, which indicated that the inhibition mechanism of these substances may not be a general property of these emulsifiers with respect to Gram-positive bacteria. It is notable that in most cases, the inhibitory action of emulsifiers increased the lag time of sensitive microbes but after time ranging from 1 to 3 days, growth resumed and the slope of growth curves in the exponential phase was comparable to that of unsupplemented cultures. These observations suggest that emulsifiers inhibit entry into active cell division by mechanisms that remain unclear. Monoculturing was informative in determining responses by different species and the levels of SSL tolerated, however, as these species were studied outside of their natural habitat, we cannot draw conclusions about how they might respond *in vivo*. It is possible that host factors (e.g., digestion) may protect sensitive species from SSL consumption.

Although this work involves only *in vitro* assays, the very low concentrations affecting the most sensitive bacteria tested, *C. clostridioforme*, *R. intestinalis*, and *B. coccoides*, suggest that consumption of SSL is likely to disturb microbiota composition *in vivo.* This speculation is supported by studies examining the structurally related compound sodium lauroyl lactylate that has been used to decrease intestinal mucosal damage and mortality caused by the enteric pathogen *Clostridium perfringens* in poultry (Lensing et al., 2010), suggesting that SSL may have an inhibitory effect on Clostridia *in vivo*. The authors concluded that a concentration over 0.15% effectively inhibited *C. perfringens* (Lensing et al., 2010), a concentration below the FDA limit for SSL in human food products, and above the concentration of SSL tested here. Another study evaluated an emulsifier mix of SSL (80%)/Tween20 (20%) as a poultry feed additive and determined that the poultry grew equally well on a low energy diet when the emulsifier mix (0.05%) was added. Although the composition of intestinal microbiota was not sequenced, *E. coli* and *Lactobacilli* populations were enumerated and no difference was seen between emulsifier and control diet, which is consistent with our *in vitro* data (Serpunja and Kim, 2019). Detailed evaluation of the ability of emulsifiers to compensate for reduced calorie intake was not established, but this could be due to altered levels of bacterial fermentation products. Propionate supplied in food has been shown to initiate metabolic events leading to increased glucagon levels in humans, and chronic consumption lead to weight gain and insulin resistance in mice (Tirosh et al., 2019).

The bacterial fermentation product butyrate is a key metabolite in human health that has been suggested to be protective against colorectal cancer (Wang et al., 2009; O’Keefe et al., 2015), inflammatory bowel disease (Segain et al., 2000) and other diseases associated a Western lifestyle (Baxter et al., 2019). Butyrate metabolism by colonocytes consumes oxygen, which facilitates maintenance of a hypoxic state of the intestinal epithelium and provides a favorable environment for anaerobic bacteria. Disruption of this positive feedback loop allows for the expansion of pathogenic facultative anaerobes, e.g., *Salmonella*, as demonstrated by the effect of antibiotic-induced depletion of Clostridia in mice that resulted in increased amounts of available oxygen in the gut (Rivera-Chavez et al., 2016). Hypoxia inducible factor, a regulator of gut barrier integrity, is dependent on the state of physiological hypoxia and thereby link butyrate consumption to intestinal barrier integrity (Peng et al., 2009; Kelly et al., 2015). These findings provide a framework to interpret our *in vitro* results that predict that emulsifiers bearing a stearate group, may reduce butyrate producing microbes that in turn may increase gut permeability of the host. Our findings that SSL increases microbial proinflammatory antigens such as LPS and flagellins may become systemic as the result of dysfunctional barrier integrity. Our results indicate that the dietary emulsifiers GMS, PGMS, and SSL have potentially detrimental effects on gut microbiota that may be coupled to disruptions in immune homeostasis facilitated by increased gut permeability. The pharmaceutical industry have studied and used emulsifiers in drug formulations due to its ability to increase gut epithelium permeability, to increase drug absorption (Nanjwade et al., 2011). It is unclear whether these effects are dependent on gut microbiota or rather reflect a direct effect of emulsifiers on gut epithelium barrier function. Taken together, our results suggest that emulsifiers may be a component of the negative influence of Western diet on intestinal microbiota and human health and substantiate additional studies *in vivo* to confirm these findings.

## Data Availability Statement

The 16S rRNA sequence datasets generated for this study can be found in the NCBI SRA accession: PRJNA600537.

## Ethics Statement

Twelve healthy human subjects who each provided written informed consent donated stool samples collected in stool hats (Thermo Fisher Scientific). The Institutional Review Board at Sanford Burnham Prebys Medical Discovery Institute approved the protocol (IRB-2014-020).

## Author Contributions

LE performed all bacterial and reporter cell culture experiments, and analyzed all data. RL and DC assisted with the aforementioned experiments. LE and SP wrote the manuscript. JZ analyzed culture DNA targeting *Escherichia* and the presence of butyrate genes by PCR. AC supported the project and contributed to study design. DR performed the bacterial genome reconstruction for butyrate and propionate pathways and calculation of SCFA production capabilities for metagenomic samples. DS performed the GC-MS analysis of SCFAs. SP supported the project.

## Conflict of Interest

The authors declare that the research was conducted in the absence of any commercial or financial relationships that could be construed as a potential conflict of interest.
