# Supplementary figures and images for: Dietary Emulsifier Sodium Stearoyl Lactylate Alters Gut Microbiota in vitro and Inhibits Bacterial Butyrate Producers
Source: Front Microbiol. 2020 May 15;11:892. doi: 10.3389/fmicb.2020.00892 (PMC7243350; doi:10.3389/fmicb.2020.00892)

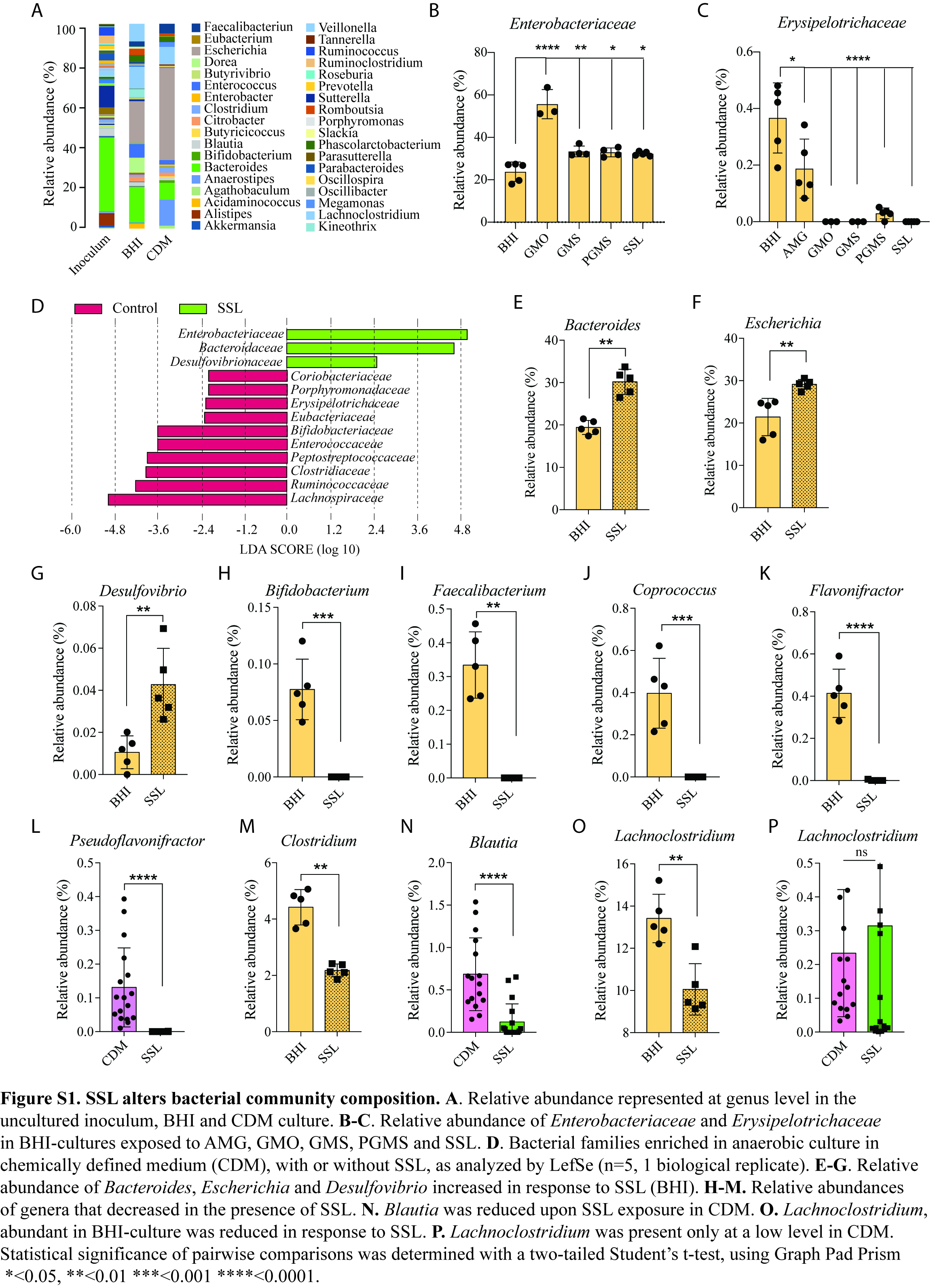

Supplement: Supplementary file 1 [file Image_1.TIF]

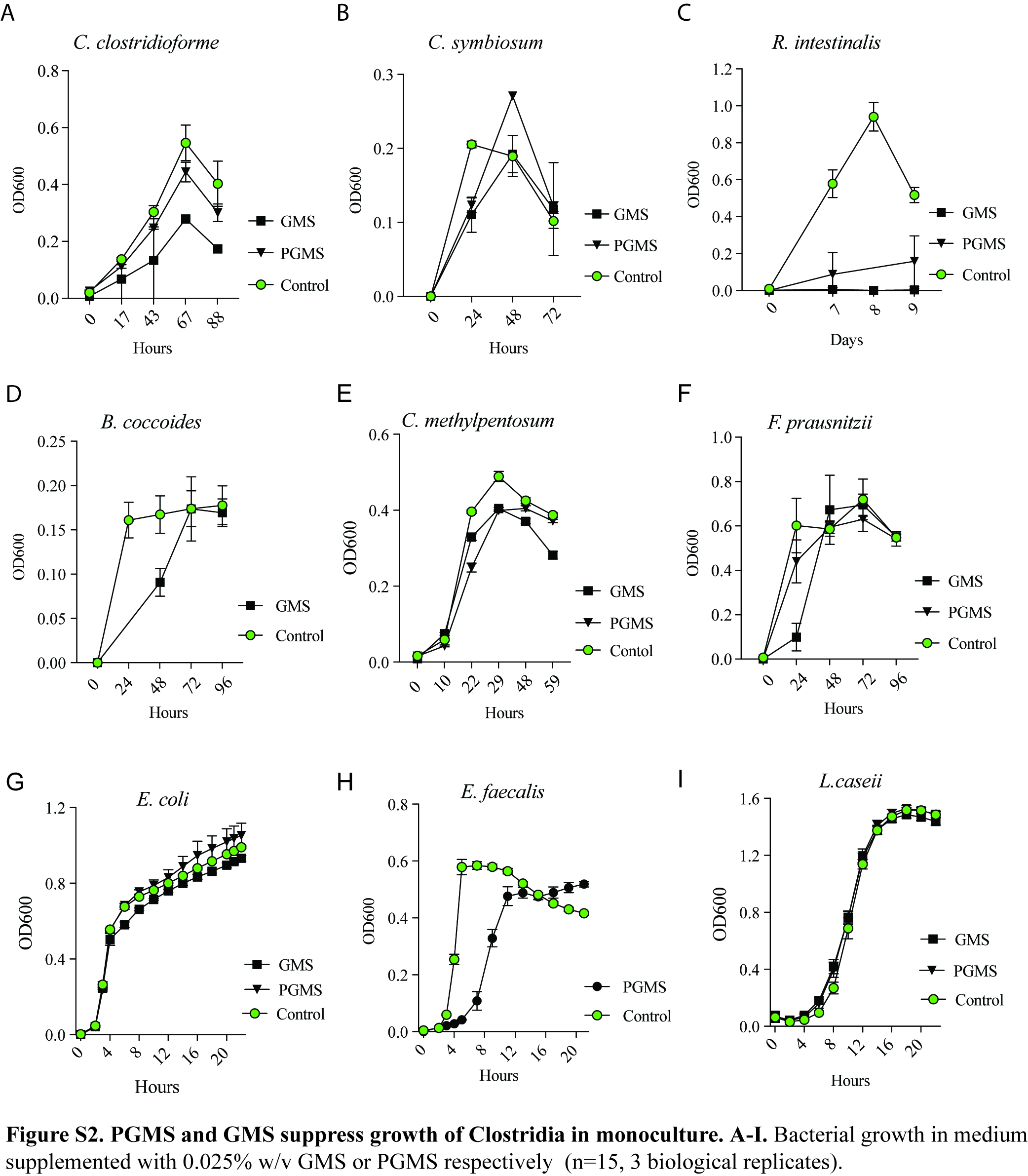

Supplement: Supplementary file 2 [file Image_2.TIF]

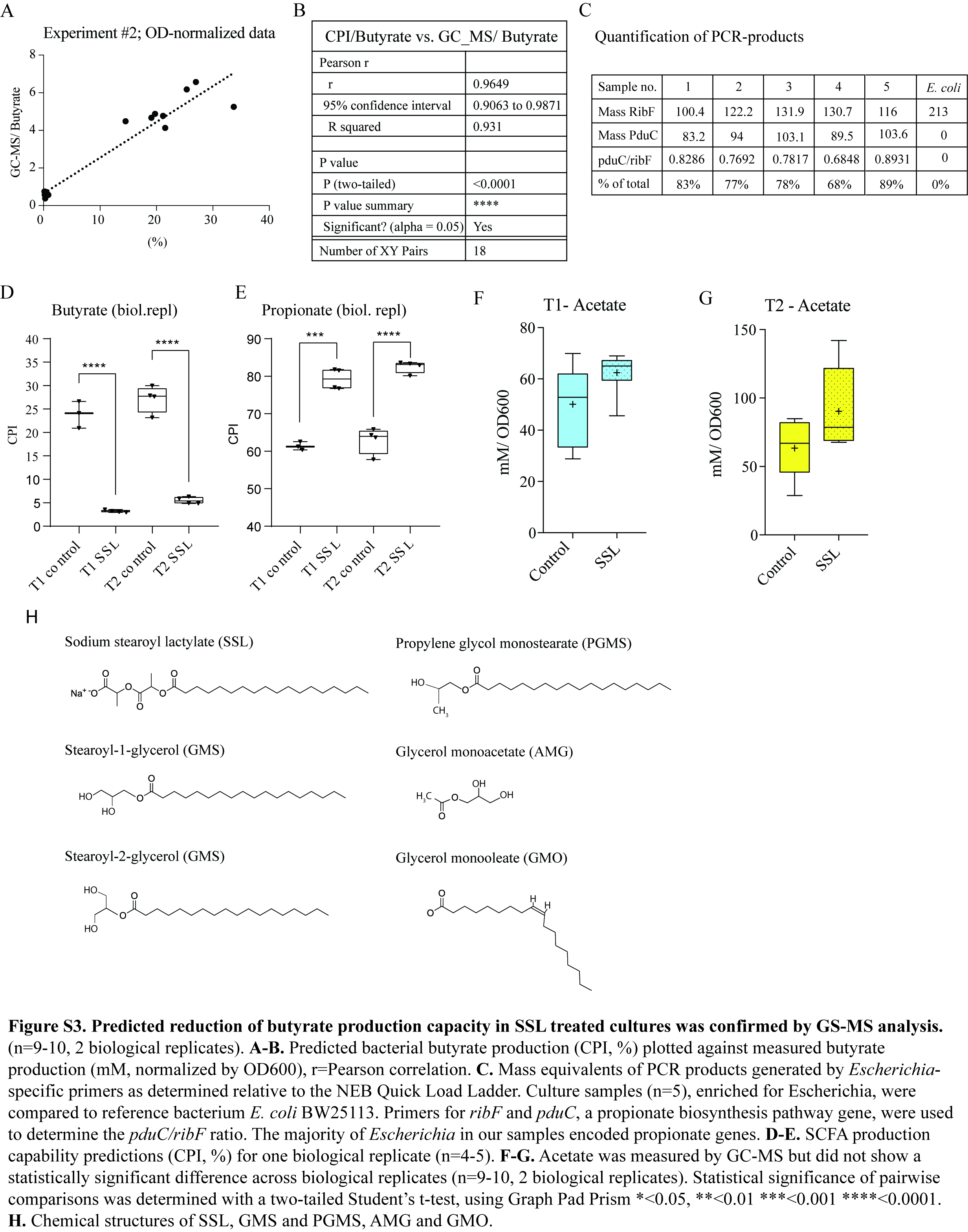

Supplement: Supplementary file 3 [file Image_3.TIF]

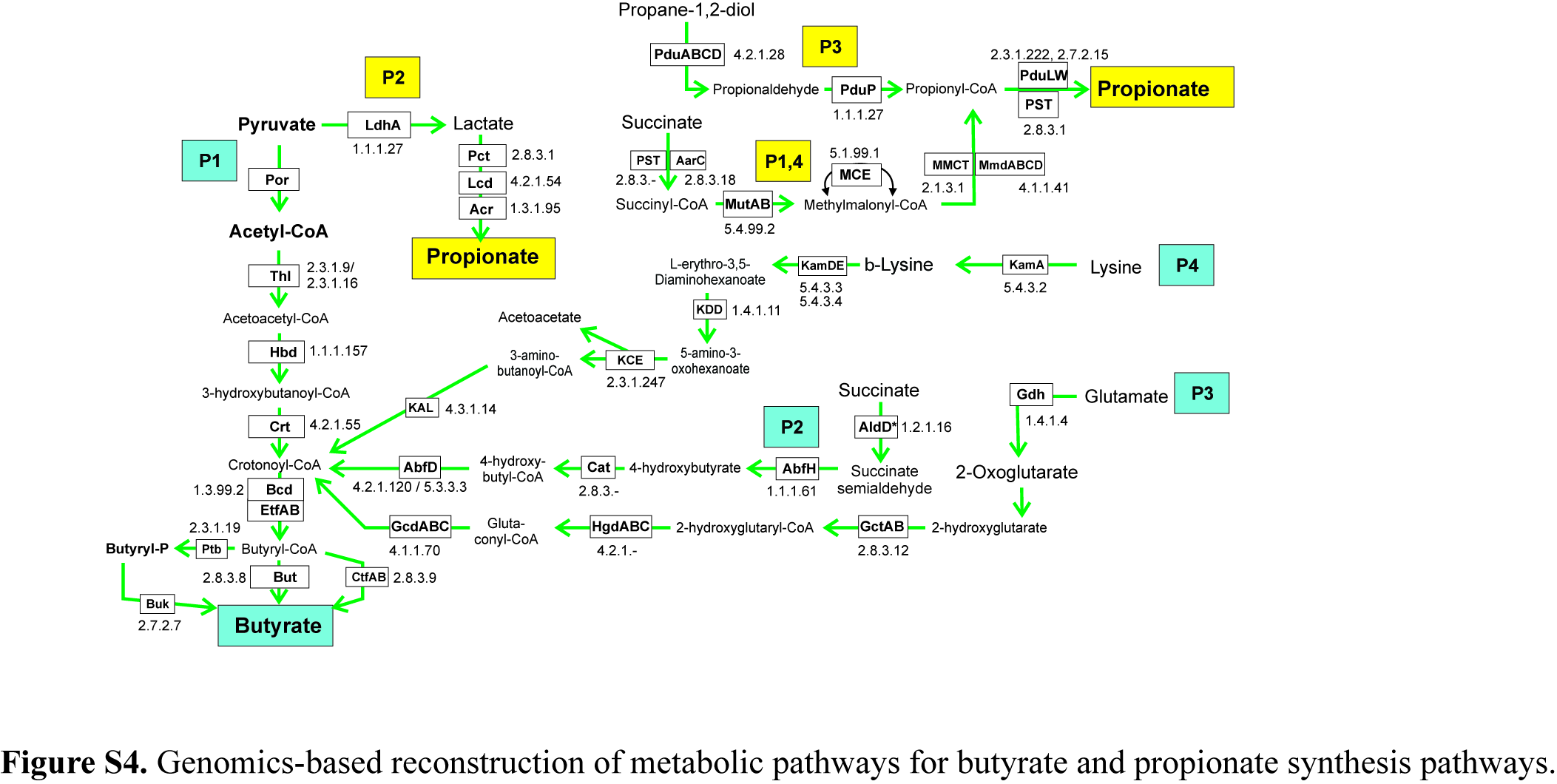

Supplement: Supplementary file 4 [file Image_4.TIF]
